# Malignancy Rates in Thyroid Imaging Reporting and Data System Category 3 (TI-RADS 3) Thyroid Nodules: A Retrospective Study

**DOI:** 10.7759/cureus.48705

**Published:** 2023-11-12

**Authors:** Renad AlSubaie, Abdullah Al Amer, Omar A Al Najjar, Kawthar Alali, Saleh Al Makhaytah, Mohammed Al Amer, Qasem M AlAlwan, Shaden S Almousa

**Affiliations:** 1 Medicine and Surgery, King Faisal University, Al Ahsa, SAU; 2 Medicine and Surgery, King Faisal University, Hofuf, SAU; 3 Oncology, Hamad Al-Jabr Oncology Center, Al Ahsa, SAU; 4 Department of Radiology, King Fahd Hospital Hofuf, Al Hofuf, SAU; 5 Medical Imaging, King Faisal University, Al Ahsa, SAU

**Keywords:** oncology, malignancy rate, ti-rads 3, thyroid nodules, radiology

## Abstract

Introduction

Thyroid cancer, one of the most frequently diagnosed endocrine malignancies, has witnessed a discernible global surge, predominantly among young adults. The etiological spectrum of thyroid cancer ranges from genetic mutations to environmental triggers. The early and precise detection of thyroid nodules (TNs) is crucial, given their latent potential for malignancy. Thyroid Imaging Reporting and Data System (TI-RADS) is an evidence-based stratification tool designed to standardize the assessment of TNs. Within this system, nodules categorized as TI-RADS 3 present an intermediate risk of malignancy, thereby necessitating meticulous evaluation. The objective of this study is to investigate the rates of cancer within thyroid nodules classified as TI-RADS 3, to determine the accuracy and effectiveness of the TI-RADS classification system in predicting malignancy at this intermediate-risk level, and to improve the diagnostic process and management strategies for these nodules.

Methods

A retrospective study was carried out on patients diagnosed with TI-RADS-3 thyroid nodules at King Fahad Hospital, Al-Hufof, between January 2019 and May 2023. Data were extracted from electronic medical records and encompassed patient demographics, and clinical and pathological details. Statistical analyses were performed using SPSS software version 27.0.1 (IBM Corp., Armonk, NY) examining the relationship between clinical characteristics and biopsy outcomes.

Results

The study involved 162 participants, mostly females (82.1%), with a median age of 43 years. Thyroid nodule analysis revealed 92.0% benign and 8.0% malignant cases, with the most common nodule size ranging from 2 to 2.4 cm. No significant correlation was found between clinical characteristics and biopsy results, indicating neither age nor gender significantly predicts malignancy in thyroid nodules within this cohort.

Conclusion

The majority of TI-RADS 3 nodules at King Fahad Hospital were benign. Yet, relying solely on TI-RADS for clinical decisions is not advised. An integrated approach, encompassing TI-RADS gradings and other nodule features, is essential for balanced patient management between intervention and observation.

## Introduction

Thyroid nodules, largely benign growths, are commonly observed within the thyroid gland. These nodules manifest in a significant proportion of adults, with prevalence estimates ranging between 20% and 76%. A notable characteristic is their augmented incidence with advancing age, implying a potential association between age-specific factors and nodule genesis [1]. While most thyroid nodules are benign and asymptomatic, the possibility of malignancy instills a need for careful scrutiny in their evaluation, given the serious implications of thyroid cancer.

The incidence of thyroid cancer has been climbing steadily, a trend that is particularly pronounced among younger adults. In the United States, for instance, thyroid cancer stands as the most prevalent cancer in men aged 30 to 39 and the second most common in women of the same age group [2]. This evolving pattern prompts inquiries regarding environmental, genetic, or diagnostic contributors to this rise.

Ultrasonography has emerged as a pivotal diagnostic modality in the identification and characterization of thyroid nodules, largely replacing more invasive procedures for initial assessments. High-resolution ultrasound, with its non-invasiveness and detailed imaging capability, is instrumental in guiding the management of these nodules, particularly in determining the necessity for fine needle aspiration (FNA) to differentiate between benign and malignant lesions [3,4]. Despite the sophistication of this technology, the interpretation of ultrasound images remains subject to clinician judgment, which carries a risk of variability and inconsistency [5].

Seeking improved diagnostic precision and uniformity, the Thyroid Imaging Reporting and Data System (TI-RADS) offers a systematic classification for gauging the potential malignancy of thyroid nodules based on ultrasound observations. The scale ranges from TI-RADS 1, denoting benign nodules, to TI-RADS 5, signifying a high malignancy probability. Distinctively positioned within this range is TI-RADS 3, designating nodules with an ambiguous or intermediate malignancy risk [6].

Nodules classified under TI-RADS 3 possess distinctive imaging characteristics. Typically, they manifest features indicative of a low to moderate suspicion of malignancy. This could encompass attributes such as a solid or largely solid composition, an iso-echoic or mildly hyperechoic appearance, well-outlined margins, and a conspicuous absence of any overtly suspicious or malignant traits. Yet, the enigma surrounding TI-RADS 3 nodules persists. While their imaging traits lean toward benignity, they are not unequivocally benign. A residual, albeit reduced, potential for malignancy shadows these nodules [7].

In this study, we delve into the enigmatic nature of TI-RADS 3 nodules, which, due to their intermediate-risk designation, demand a nuanced understanding to refine management strategies. We investigate the malignancy rates in nodules categorized as TI-RADS 3, aiming to illuminate the risk profile of this ambiguous group. Our research is poised to provide a more robust epidemiological understanding of thyroid nodules and their malignancy potential, with an emphasis on improving the diagnostic accuracy and consistency afforded by TI-RADS. Ultimately, the findings from this study seek to contribute significantly to the ongoing conversation on thyroid nodule assessment and management, ensuring better outcomes for patients encountering this common clinical scenario.

## Materials and methods

Study design and setting

This retrospective study was carried out at the King Fahad Hospital in Al Hofuf, Al Ahsa, Saudi Arabia. The hospital serves as a major healthcare provider in the region, equipped with state-of-the-art medical facilities and experienced healthcare professionals.

Study population and period

Patients who presented with thyroid nodules classified under TI-RADS 3 from January 1, 2019, to May 20, 2023, constituted the target population for this study. A total duration of four years and nearly five months provided a substantial timeframe for collecting a comprehensive set of data.

Data collection

Data were meticulously retrieved from electronic medical records and imaging reports available in the hospital's database. These data included extensive details such as patient demographics (age, gender), clinical history (symptoms, previous medical conditions), laboratory findings (hormone levels), imaging reports (ultrasound characteristics, nodule size), and pathological reports (histopathology results).

Sample size and inclusion criteria

The sample size for our study was determined through a power analysis, which was conducted prior to data collection to ensure a robust study design. We aimed for a power of 80% to detect a medium effect size (Cohen's d of 0.5) with a significance level of 0.05. These parameters were chosen based on the expected clinical relevance of differences in malignancy rates among TI-RADS 3 nodules, informed by previous literature and expert consultation. The analysis suggested that a minimum of 157 participants would be necessary to confidently identify the presence of clinically significant findings. To account for potential dropouts and missing data, we increased the sample to 162 participants. The inclusion criteria for the study were strictly followed, encompassing patients who were 18 years of age or older and had undergone a fine-needle aspiration biopsy (FNAB) or surgical excision of the thyroid nodule.

Ethical considerations

Ethical approval was secured from the Institutional Review Board (IRB) at King Fahad Hospital, with the reference number H-05-HS-065. All patient data was anonymized and handled confidentially to uphold the privacy and rights of the participants involved in the study.

Statistical analysis

Descriptive Analysis

Initial analysis of the data involved descriptive statistics to outline the sociodemographic and clinical characteristics of the study population. Frequencies and percentages were calculated for categorical variables, while medians and interquartile ranges were reported for continuous variables such as age. The choice of medians and interquartile ranges was based on the assessment of the data’s distribution, where the Kolmogorov-Smirnov test and Q-Q plots indicated a non-normal distribution (p<0.05).

Inferential Analysis

Subsequent to the descriptive analysis, inferential statistical methods were employed to discern patterns and associations within the data. A comparative analysis between patients with benign and malignant biopsy results was conducted. Fisher's exact test was utilized for categorical variables, ensuring a robust comparison irrespective of sample sizes. For the age variable, being continuous but non-normally distributed, the Mann-Whitney U test was applied to compare the two groups.

Significance and Software

A p-value threshold of 0.05 was established to determine statistical significance, correlating to a 95% confidence interval for the results obtained. All statistical analyses were conducted using the SPSS software, version 27.0.1 (IBM Corp., Armonk, NY) ensuring accurate and reliable results.

## Results

The study included 162 participants with thyroid nodules that met the inclusion criteria. The median age of these participants was 43 years, with an interquartile range of 37 to 45 years. The age distribution analysis revealed that the largest group of participants was in the 41-45 age range, accounting for 37.7% of the total. Among these, 82.1% identified as female, and 17.9% were male (Table 1).

**Table 1:** Overall sociodemographic characteristics of the participants (N=162) Mdn: Median; IQR: Interquartile range

		Mdn / N	IQR / %
Age		43	37-45
Age (Range)	18-25	10	6.2%
	26-30	6	3.7%
	31-35	15	9.3%
	36-40	33	20.4%
	41-45	61	37.7%
	46-50	20	12.3%
	51-55	11	6.8%
	56-60	6	3.7%
Gender	Female	133	82.1%
	Male	29	17.9%
Total		162	100.0%

**Figure 1:**
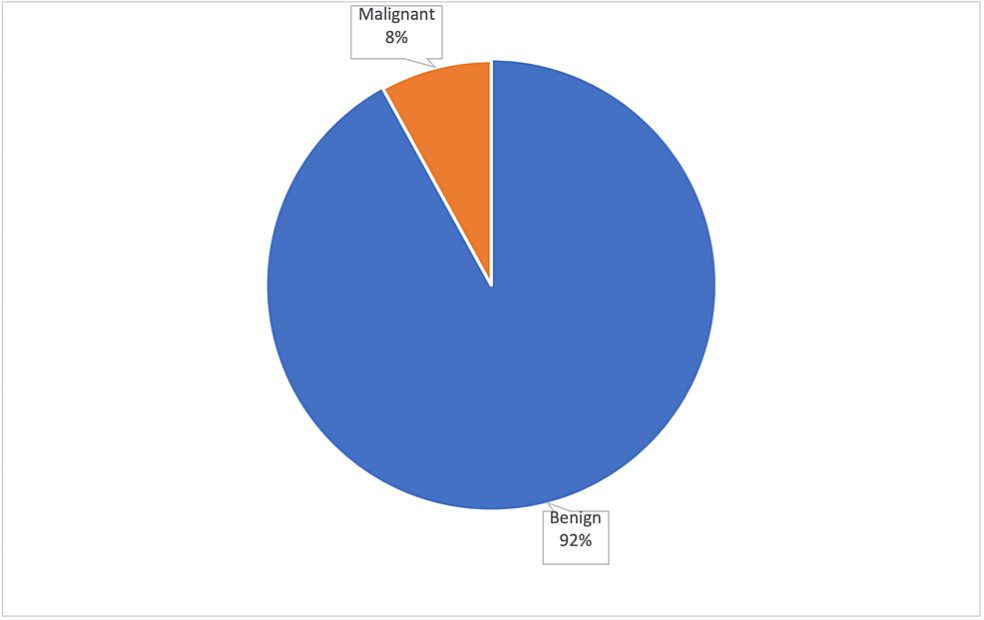
Proportion of malignant and non-malignant thyroid nodules

Most participants had enlarged lymph nodes, representing 73.5% of the total. The size of the thyroid nodules varied, with the most common size range being 2 to 2.4 cm, accounting for 14.2% of cases. Regarding biopsy results, a significant 92.0% showed benign findings, with the remaining 8.0% indicating malignancies (95% CI for proportion benign: 86.2% to 95.8%) (Figure 1). A detailed breakdown of the biopsy results, including Bethesda categories, is available in Table 2.

**Table 2:** Investigations and outcomes of the participants (N=162) TN: Thyroid nodule

		N	%
Thyroid disease	Goiter	17	10.5%
	Hashimoto's Thyroiditis	2	1.2%
	Lymphocytic Thyroiditis	3	1.9%
	Thyroid Nodules	29	17.9%
	Toxic multinodular goiter	16	9.9%
Biopsy Recommended?	For clinical correlation	65	40.1%
	No	33	20.4%
	Yes	64	39.5%
Enlarged Lymph Nodes	No	43	26.5%
	Yes	119	73.5%
Size of TN (Range)	0.1-0.4cm	9	5.6%
	0.5-0.9cm	10	6.2%
	1-1.4cm	10	6.2%
	1.5-1.9cm	12	7.4%
	2-2.4cm	23	14.2%
	2.5-2.9cm	19	11.7%
	3-3.4cm	14	8.6%
	3.5-3.9cm	12	7.4%
	4-4.4cm	9	5.6%
	4.5-4.9cm	5	3.1%
	5-5.4cm	10	6.2%
	5.5-6cm	5	3.1%
Biopsy Result	Benign	149	92.0%
	Malignant	13	8.0%
Biopsy Results Detailed	Benign	80	49.4%
	Bethesda Category I	9	5.6%
	Bethesda Category II	25	15.4%
	Bethesda Category III	33	20.4%
	Bethesda Category IV	5	3.1%
	Malignant	9	5.6%

Comparing participants with benign and malignant biopsy results, the median age for those with benign findings was 43 years (IQR: 37-45), and the effect size for age as a predictor of biopsy results was d=0.15 (95% CI for effect size: -0.25 to 0.55), indicating a small and not statistically significant difference. In contrast, for those with malignant results, the median age was 42 years (IQR: 38-47), and the malignancy rate was slightly higher in females (9.8%), but this difference was also not statistically significant (p = 0.127). The effect size for gender as a predictor was d=0.20 (95% CI for effect size: -0.30 to 0.70). Interestingly, there were no statistically significant associations between the clinical characteristics examined-specifically, thyroid disease, biopsy recommendations, enlarged lymph nodes, and thyroid nodule size-and biopsy results (Table 3).

**Table 3:** Association of biopsy results with sociodemographic characteristics and investigations Independent Samples Mann-Whitney U Test, Fischer's Exact Test *p<0.05, Significant; TN: Thyroid nodule

Benign (N=149)				Malignant (N=13)		P
		Mdn / N	IQR / Row %	Mdn / N	IQR / Row %	value^U,F^
Age		43	37-45	42	38-47	0.632
Gender	Female	120	90.2%	13	9.8%	0.127
	Male	29	100.0%	0	0.0%	
Thyroid disease	Unknown	87	91.6%	8	8.4%	0.688
	Goiter	16	94.1%	1	5.9%	
	Hashimoto's Thyroiditis	2	100.0%	0	0.0%	
	Lymphocytic Thyroiditis	3	100.0%	0	0.0%	
	Thyroid Nodules	25	86.2%	4	13.8%	
	Toxic multinodular goiter	16	100.0%	0	0.0%	
Biopsy Recommended?	For clinical correlation	61	93.8%	4	6.2%	0.653
	No	31	93.9%	2	6.1%	
	Yes	57	89.1%	7	10.9%	
Enlarged Lymph Nodes	No	40	93.0%	3	7.0%	1.000
	Yes	109	91.6%	10	8.4%	
Size of TN (Range)	0.1-0.4cm	9	100.0%	0	0.0%	0.351
	0.5-0.9cm	10	100.0%	0	0.0%	
	1-1.4cm	9	90.0%	1	10.0%	
	1.5-1.9cm	12	100.0%	0	0.0%	
	2-2.4cm	21	91.3%	2	8.7%	
	2.5-2.9cm	19	100.0%	0	0.0%	
	3-3.4cm	12	85.7%	2	14.3%	
	3.5-3.9cm	10	83.3%	2	16.7%	
	4-4.4cm	8	88.9%	1	11.1%	
	4.5-4.9cm	5	100.0%	0	0.0%	
	5-5.4cm	7	70.0%	3	30.0%	
	5.5-6cm	5	100.0%	0	0.0%	

## Discussion

In the field of thyroid nodule diagnosis, TI-RADS 1 and 2 predominantly indicate benign cases, while TI-RADS 4 and 5 strongly suggest potential malignancies. In this landscape, TI-RADS 3 occupies an ambiguous position between these clear categories [8,9]. Our study addresses the uncertainty surrounding TI-RADS 3 nodules, seeking clarity on whether they tend towards being cancerous or if they can be confidently classified as benign.

The biopsy outcomes for TI-RADS 3 nodules predominantly suggest a benign pathology, with 92.0% demonstrating non-malignant characteristics. In contrast, malignancy was observed in only 8.0% of nodules (Figure 1). This finding diverges markedly from another investigation, which reported a higher proportion-33% of TR3 nodules being malignant or suspicious of malignancy [10,11]. The significant discrepancy between these results may be attributed to differences in sample sizes; the referenced study evaluated a larger cohort of 900 nodules. Our study thus affirms the generally benign prognosis of TI-RADS 3 nodules, yet it also emphasizes the importance of vigilant follow-up and precise management to promptly address any malignant transformations.

The presence of enlarged lymph nodes was identified in 119 out of 162 (73.5%) patients categorized as TI-RADS 3, as indicated in Table 2. Interestingly, Table 3 connects many malignant biopsy outcomes with these enlarged lymph nodes. This connection hints at a possible relationship between enlarged lymph nodes and malignancy within the TI-RADS 3 group, suggesting a deeper investigation could be valuable for clinical evaluations.

Thyroid malignancies have a higher detection rate in females. This observation is supported by comprehensive research, indicating that around 75% of documented thyroid cancer cases are found in women [12]. Our data mirrors this pattern: 82.1% of participants presenting with nodules were female (Table 1). An evaluation of our entire FNAB patient cohort revealed a dominant presence of malignancies among female participants (Table 3). This trend can be attributed to the varied impact of female sex hormones on the body's systems. Changes in these hormones during menstrual cycles and pregnancy might lead to gender-based disparities in several thyroid cancer types, especially papillary thyroid cancer [13]. The peak incidence of papillary thyroid cancer coincides with the typical age for menopause (40-49 years), which aligns with our findings in Table 3. While many studies probe the potential connection between thyroid carcinoma and menopause, the definitive role of menopause in thyroid cancer remains elusive [14].

Furthermore, several risk factors are associated with thyroid malignancy, and among them, radiation exposure emerges as a noteworthy concern, particularly when it affects the head and neck area. Ionizing radiation can disrupt DNA, resulting in breakages and the occurrence of multiple mutations. Some of these mutations kickstart the process of carcinogenesis, establishing radiation exposure as a commonly acknowledged risk factor for thyroid carcinoma. Additionally, the role of chromosomal alterations holds significance in the progression of thyroid cancer [15].

In this study, the Bethesda classification was employed for the pathological assessment of FNAB. The Bethesda classification system divides thyroid biopsy results into six categories, each of which contains specific criteria that determine whether a thyroid nodule is expected to be benign or malignant. Every category correlates with a particular risk of malignancy and has its own method of management and intervention [16]. Figure 2 illustrates that almost half of the biopsy results for thyroid nodules were benign with 49.40%. The second highest occurrence was placed under Bethesda category III (AUS) at 20.40%, followed by 15.40% of the results under Bethesda category II. 5.60% of the results indicated malignancy, while only 0.60% were noted as Bethesda category VI. These results could be justified by the fact that most of the tumors under TI-RAD 3 have smooth borders and are regular in shape, which are characteristic features of benign tumors. On the other hand, if the tumor is solid, irregular, or hypoechoic, there are higher probabilities of malignancy.

In terms of ultrasound evaluation, thyroid nodules have five core assessment features: composition, echogenicity, shape, margin, and echogenic foci. Each is scored and combined to gauge the overall risk level [17]. Our findings brought forward some distinct patterns in TI-RADS 3 patients: 41.4% exhibited hyperechoic/isoechoic traits. Furthermore, the nodule composition varied widely, with 21.6% (35 cases) being almost wholly solid, and 34.6% (56 cases) presenting a combined cystic and solid structure (Table 2).

It is important to note the limitations of our study. The single-center design may limit the generalizability of our results across different populations and healthcare settings. Additionally, male participants were underrepresented, making up only 17.9% of our cohort, which may affect the applicability of our findings to both genders. Also, the use of electronic medical records carries the risk of incomplete or inaccurate data.

## Conclusions

Our study confirmed that TI-RADS 3 thyroid nodules mainly display benign features. This result supports the credibility and utility of the TI-RADS system in estimating the likelihood of malignancy in thyroid nodules, thus improving diagnostic precision. By enhancing diagnostic certainty, we illuminate pathways for diminishing healthcare burdens and costs through the judicious use of FNAB, predicated on accurate risk stratification. Future studies should expand beyond a single-center approach to include diverse populations across multiple healthcare settings, ensuring broader applicability of results. Efforts to balance gender representation are crucial for comprehensive gender-specific analyses. Incorporating robust data validation measures and possibly integrating genetic markers could further refine the predictive accuracy for thyroid nodule malignancy. Longitudinal designs are also recommended to observe nodule progression over time, providing a more dynamic understanding of TI-RADS 3 outcomes and informing potential updates to assessment guidelines and management strategies.
